# Differential regulation of ribogenesis genes throughout oogenesis in thicklip grey mullets, *Chelon labrosus*, preparing the maternal contribution to embryo development

**DOI:** 10.1371/journal.pone.0356468

**Published:** 2026-09-18

**Authors:** Joyanta Bir, Oihane Diaz de Cerio, Maren Ortiz-Zarragoitia, Ibon Cancio

**Affiliations:** 1 CBET+ One Health Research Group, Dept. of Zoology & Animal Cell Biology, Centre for Experimental Marine Biology & Biotechnology (PiE-EHU), University of the Basque Country (EHU), Plentzia, Spain; 2 Fisheries and Marine Resources Technology Discipline, School of Life Sciences, Khulna University, Khulna, Bangladesh; Shanghai Ocean University, CHINA

## Abstract

Oocytes in teleosts undergo complex changes during oogenesis, including the accumulation of ribosomal RNAs and tRNAs needed to support protein synthesis during early embryogenesis. Previous studies show that high levels of 5S rRNA and tRNAs in perinucleolar previtellogenic (PV) oocytes, and the easily calculated 5S rRNA/18S rRNA index, reliably distinguish testes from ovaries, especially when ovaries are in PV. Building on this, we examined whether the tRNA/5.8S rRNA index follows a similar pattern during oogenesis in thicklip grey mullet *Chelon labrosus*, a species with synchronous ovarian development. The index values revealed very high tRNA levels during previtellogenesis, when only perinucleolar oocytes are present, tRNA levels being gradually met by 5.8S rRNA ones during secondary growth towards vitellogenesis. Transcription of 5S rRNA and tRNAs by RNAP-III is regulated by general transcription factors (Gtf3a, b, and c) and the inhibitor Maf1. We analyzed ovarian transcription patterns of Gtf3b-related genes (*brf1a, brf1b, brf2, bdp1*, and *tbpl2*), comparing them with previously described *gtf3aa* and *gtf3ab*, which act specifically in 5S rRNA transcription. We also quantified transcript levels of *maf1* and *dap1b*, the latter involved in keeping ribosomes dormant until fertilization. Most Gtf3b-related genes, as well as *maf1* and *dap1b*, showed higher transcription in ovaries than in testes and intersex testes. Transcript levels peaked in PV ovaries and declined in cortical alveoli and late-vitellogenic stages. *brf1a* and *tbpl2* also differentiated intersex testes from normal testes. These observations reinforce the hypothesis that primary oocyte differentiation is marked by a strong activation of RNAP-III activity for ribogenesis.

## 1. Introduction

During teleost gametogenesis, gametes experience extensive molecular transformations that in the case of oocytes facilitate meiosis and growth through the incorporation of crucial nutrients and biomolecules, including tRNAs and RNAs, all vital for subsequent embryonic development after fertilization [1–3]. 5S rRNA for instance is highly accumulated in previtellogenic oocytes, while larger rRNAs produced from 45S rRNA accumulate during oocyte secondary maturation in the progression from cortical alveoli to full maturity. In this way, the strong accumulation of 5S rRNA in oocytes serves as marker of oocytes while the ratio 5S to 45S rRNA maturation transcripts serves as a descriptor of progress through oogenesis and oocyte maturation [1,4]. In the same way, as a molecule accumulated in oocytes, 5S rRNA serves as marker of intersex condition in testes of males environmentally exposed to xenoestrogens [1,5]. The 5S/18S rRNA index that was established by our research group numerically, indicates the stage of development of the ovary of a teleost fish [1,5]. This differential production and accumulation of rRNA molecules reflects changes in gene transcription regulation within developing oocytes and their surrounding cells [6].

Eukaryotic gene transcription relies necessarily on RNA polymerases I, II, and III (RNAP-I, II, and III) [7,8]. RNAP-I synthesizes the large polycistronic 45S ribosomal RNA, a precursor to 5.8S, 18S, and 28S rRNAs. RNAP-II produces all messenger RNAs (mRNAs) and various non-coding RNAs, while RNAP-III generates small non-coding RNAs, primarily tRNAs, 5S rRNA, 7SL RNA and U6 snoRNA [7–9]. This process involves an intricate regulation of RNAP-III activity by specific general transcription factors (TFIIIA, TFIIIB, and TFIIIC) [8,10]. In teleost fish genomes, the genes encoding the proteins that form these transcription factors have undergone duplication events [3,11]. One of these transcription factors is coded by one single gene that has suffered duplication and subfunctionalization in teleosts, *gtf3ab* acting as a maternal gene exclusively and highly transcribed in oocytes during previtellogenesis [3,5]. This transcription accompanies the strong transcription of 5S rRNA, as TFIIIB is exclusively needed for expression of 5S rRNA coding genes, and also for cytosolic stockpiling of the transcript itself [3,12].

The TFIIIB complex is composed of three subunits: Brf1 (or Brf2, in the case of some specific gene promoters), Bdp1, and a TATA-box binding protein (TBP) [9,13]. *brf1* and *bdp1* are duplicated in the genomes of some teleost groups while three different TBP paralogs exist in all vertebrates [11]. Additionally, Maf1 is a RNAP-III key negative regulatory peptide that binds to the enzyme in its dephosphorylated state inhibiting its transcriptional activity [14,15]. When phosphorylated under favourable conditions, Maf1 dissociates from RNAP-III, allowing transcription initiation [16,17]. Ribogenesis is a highly energy consuming process and it seems that in oocytes ribosomes would be abundantly accumulated as maternal legacy to support rapid protein synthesis during early embryo development [18]. These ribosomes remain in a dormant state until necessary during early embryogenesis [19,20]. The newly identified ribosome-associated protein Dap1b occupies the ribosomal peptide exit tunnel and contributes to translational repression by interacting with eIF5A at the P-site in dormant 80S ribosomes [20].

The thicklip grey mullet (*Chelon labrosus*) has recently emerged as a promising candidate for aquaculture diversification in Europe, particularly in Spain, owing to its availability, omnivorous diet, and euryhaline adaptability [21–23]. It belongs to the Mugilid family, which includes approximately 25 genera and 80 species, broadly distributed throughout the Northeast Atlantic Ocean, Mediterranean, and Black Seas [24–26]. This euryhaline species, known for high residency in coastal systems, utilizes estuaries as nurseries and foraging grounds, migrating with tidal movements [24,27]. During winter (December–February), adults migrate offshore to spawn, producing pelagic eggs that hatch within 3–4 days, followed by a larval phase lasting approximately four weeks [28]. Juveniles form dense schools and migrate to shallow estuaries where they remain until maturity. The prolonged contact of those juvenile *C. labrosus* to xenoestrogens and endocrine disruptors chemicals (EDCs) makes this species an effective sentinel for assessing environmental health [25,28,29]. Exposure to xenoestrogens can disrupt gonad development, interfering with oocyte recruitment and sometimes induce intersex gonads (when oocytes develop within testes) in fish, as observed in thicklip grey mullets from polluted estuaries in the Southern Bay of Biscay [2,25,30].

There are not enough studies focused on the genes coding TFIIIB and their specific transcription regulation throughout the oogenesis process in teleosts. In this framework, the current study aimed to compare the transcription profiles of genes associated with ribogenesis throughout the oogenic cycle of thicklip grey mullets. We also investigated whether the tRNA/5.8S rRNA index exhibits the same profile as the previously established 5S rRNA/18S rRNA index during oogenesis in the synchronously developing *C. labrosus*, and whether these could serve as molecular markers for identification of oocyte maturation stages in teleost fish. The ovarian transcription patterns throughout oogenesis of genes encoding TFIIIB polypeptides (*brf1a, brf1b, brf2, bdp1,* and *tbpl2*) were compared to those of the previously characterized *gtf3aa* and *gtf3ab*, along with those of the RNAP-III inhibitor *maf1*, the sex marker genes *cyp19a1a* and *amh*, plus a ribosome dormancy associated gene *dap1b*. Essentially, we wanted to understand the dynamics of ribosome related gene transcription along teleost oogenesis.

## 2. Materials and methods

### 2.1 Ethics statement

Fish handling and procedures were approved by the UPV/EHU Ethics Committee on Animal Experimentation (CEEA by its initials in Spanish) and authorisation (Code: CEEA/337-4/2014/ORTIZ ZARRAGOITIA) granted by the provincial authorities (Provincial Council of Biscay), that includes the procedures for alleviation of suffering and sacrifice for thicklip grey mullet. The animal study is reported in accordance with ARRIVE guidelines (https://arriveguidelines.org) for animal research. All methods were performed in accordance with the relevant guidelines and regulations.

### 2.2 Study area, fish sampling and tissue collection

Thicklip grey mullet (*C. labrosus*) specimens were collected from Plentzia harbor (43°24’26.1“N, 2°56’45.8”W, Southeastern Bay of Biscay) over the period from July to November 2023, and in January 2024 to cover the whole reproductive cycle. Individuals were captured using a fishing rod, the sampled fish displaying an average total length of 38.45 ± 4.9 cm and an average weight of 785 ± 302.39 g. Females fished during these samplings were selected for this study. In addition, intersex male mullets were selected from those captured during annual monitoring campaigns (2014–2021) conducted by the Biscay Bay Environmental Biospecimen Bank (BBEBB; PiE- EHU) in the Gernika estuary (43°19′26″N, 2°40′26″W; SE Bay of Biscay) and long-term stored in liquid nitrogen tanks. Intersex individual came from different sampling years, but this was the only way to ensure enough individuals could be analysed, a total of 6 per severity group. For comparability, non-intersex testes were also selected from Gernika mullets, spermatogenic stage being the same as the one in the intersex testes. Immediately after capture, mullets were anesthetized in a water bath containing saturated ethyl-4-aminobenzoate. All handling with anaesthetic complied with institutional safety regulations and with the recognized human endpoint of concern. When fish were completely anesthetized (no opercular movement observed), fish were decapitated and each gonad was dissected for molecular and histological analyses. The portion designated for molecular studies was preserved in RNAlater® (Ambion, Life Technologies, Carlsbad, USA), snap-frozen in liquid nitrogen, and stored at −80 °C for future use. The tissue sample intended for histological examination was fixed in 10% neutral buffered formalin. For intersex mullets, *in-situ* dissection was performed, and one portion of each gonad was stored in RNAlater® before being frozen in liquid nitrogen until arrival to the laboratory.

### 2.3 Histological analysis and ovary development staging

For histological analysis gonad samples were fixed for 24 h in 10% neutral buffered formalin, then dehydrated in a graded ethanol series (70%, 90%, and 96%), and embedded in paraffin. Histological sections of 5 μm were cut using a microtome (Leica RM 2125 RT, Leica Microsystems, Nussloch, Germany) and stained with Hematoxylin-Eosin using the Leica Autostainer XL and mounted with the aid of the Leica CV 5030 workstation. Gonad histological sections were examined microscopically with an Olympus BX61 light microscope (Tokyo, Japan). The gametogenic stages of each individual were determined according to McDonough et al. (2005) [31], and defined according to the prevailing stage oocytes as previtellogenic (PV), cortical alveoli (CA), and advanced vitellogenic (AV). Based on the presence of oocytes in the testes, intersex testes were classified into two severity groups: low intersex severity (I.I. 1,2) and high intersex severity (I.I. 3,4), using the intersex index scale described by Blazer et al. (2007) [32]. From each of these organs and histological categories, six independent samples per group (except for the CA with *n = 5*) were selected for subsequent molecular analyses.

### 2.4 RNA extraction and quality check

Total RNA was extracted from 50–100 mg of *C. labrosus* tissues (ovaries, testes and intersex testes) using TRIzol Reagent® (Ambion, Life Technologies, Carlsbad, California, USA) following the manufacturer’s protocol. After extraction, RNA was resuspended in RNase/DNase-free water (30–50 µl), and stored at −80 °C to preserve the RNA integrity.

RNA concentration and quality were spectrophotometrically measured in a microplate spectrophotometer (BioTek Epoch, Agilent Technologies, Santa Clara, California, USA) followed by Nano 6000 and small RNA Assay analysis in a 2100 Bioanalyzer (Agilent Tecnologies, Santa Clara, California, USA) according to the manufacturer protocol. All samples showed A260/A280 ratios between 1.8 and 2.2, confirming their suitability for further downstream analysis.

### 2.5 Quantification of tRNA/5.8S rRNA and 5S/5.8S rRNA indices

Concentrations of tRNAs, 5S rRNA and 5.8S rRNA were obtained directly from peaks specific to each RNA sample provided by the electropherogram of the Bioanalyzer. The time-corrected peak areas for tRNAs, 5S rRNA, and 5.8S rRNA were measured and used to calculate the following ratios: tRNA/5.8S rRNA, and 5S/5.8S rRNA according to Bir et al. (2024) [33]. These ratios were then used to rank the ovaries according to their different developmental stages [4,33].

### 2.6 cDNA synthesis and quantification

First-strand cDNA was synthesized by using Affinity Script Multiple Temperature cDNA synthesis kit (Agilent Technologies, Cedar Creek, Texas, USA) in a SimpliAmp™ Thermocycler (model A24811, Applied Biosystems, Life Technologies, Carlsbad, California, USA). Each reaction was performed with up to 2 μg of total RNA in a final volume of 20 μL, resulting in a theoretical cDNA concentration of 100 ng/μL. The cDNA concentration was quantified in 96-well plates using the Quant-iT™ OliGreen® ssDNA Assay Kit (Invitrogen, Life Technologies, Eugene, Oregon, USA) in a Biotek FLx800 Fluorescence Microplate Reader (Biotek, Winooski, VT, USA). Fluorescence was measured at excitation and emission wavelengths of 485/20 nm and 528/20 nm, respectively. Actual cDNA concentrations were determined using a high-range standard curve in accordance with the manufacturer’s guidelines. Based on these calculations, the exact amount of cDNA loaded for each RT qPCR reaction was determined, and the dilution for each gene was adjusted accordingly [5].

### 2.7 Target gene transcription analyses by RT qPCR

Target gene sequences were obtained from the NCBI GenBank database (Table 1). Primer pairs of each gene were designed using Eurofins online primer design tools (https://eurofinsgenomics.eu/). The specificity of each gene was verified by conventional PCR amplification on at least two independent cDNA samples of each tissue. The amplification was run with commercial Taq DNA Polymerase recombinant Kit and 100 mM dNTP Mix (Invitrogen, Thermo Fisher Scientific, Carlsbard, California, USA) in a SimpliAmp™ Thermocycler. The PCR products were visualized in 1.5% (w/v) agarose gels stained with GreenSafe Premium (NZYtech, Lisbon, Portugal) and sequenced at the Sequencing and Genotyping Service of University of the Basque Country (SGIker-UPV/EHU), and finally the sequences analysed through Blast analysis to confirm the specificity.

**Table 1:** Specific forward (F) and reverse (R) primers sequences used for the RT qPCR analysis of target gene transcription levels in *Chelon labrosus* showing the corresponding GenBank accession numbers, amplicon size (bp), annealing temperature (T_A_), primer concentration (pMol), primer efficiency (E) and cDNA dilution factors.

Target gene	GenBank accession No.	Primer Sequence (5’-3’)	Amplicon size (bp)	T_A_ (°C)	Primer (pMol)	E (%)	dilution factor
*cyp19a1a*	EF535845.1	F: GCACCTACTTCTCCAAAGCC	105	59.8	6.25	95.18	1:5
		R: GCCAAACTGTCCAAGTCGTC					
*amh*	MH251321.1	F: CCTGCTCAACTTCCACATCG	151	59.5	6.25	91.53	1:2
		R: CCCACACTCCTTTGCTATCAC					
*gtf3aa*	PQ633379.1	F: GTCTTTCACCACAACCTTCAAC	167	58.5	6.25	99.89	1:20
		R: GCCTCTTCAGCTTCTTCCTC					
*gtf3ab*	JN257141.1	F: TGGGGGAGAGGTTGAAAAGTC	174	59.5	6.25	99.7	1:100
		R: GTCTGGTGAGTTGATAGCGAG					
*brf1a*	MW175517.1	F: GAAGGTGGAGCCAAACTTCC	156	60	6.25	102.37	1:5
		R: GTTGAGGGCCGTGTCCAGA					
*brf1b*	MW175518.1	F: CTGGATGAGGTTGAAGGAG	225	58	6.25	103.17	1:20
		R: CTGACAGAGGAAGTCCTGG					
*brf2*	MW175516.1	F: GGTGGGAGTTGTTTATCAAGAG	218	58	6.25	95.52	1:20
		R: CACAATCCAGGAATCTGCCG					
*bdp1*	PQ633376.1	F: GAGGAAGCAGAGGAAGAGG	216	59.0	12.5	108.44	1:20
		R: GGACCAGGGTTTGGAATAGG					
*tbpl2*	PQ633377.1	F: GACCCCCATGACCCCCAT	240	59.5	6.25	95.83	1:5
		R: CTCTTGGCTCCAGTACAGAC					
*maf1b*	PQ633380.1	F: GAGTTCAGCCGAGAGCCAAG	272	60.5	12.5	100.36	1:2
		R: CACTGACTGAGCGGCATGTG					
*dap1b*	PQ633378.1	F: GCCTCTTAGTCTCCTTCTCT	159	57.5	6.25	91.61	1:20
		R: GGTGCAACAACTGTCCAAATC					

Transcription levels of target genes were quantified using FastStart Universal SYBR Green Master (Rox) (Roche, Mannheim, Germany) with a ViiA-7 Real-Time PCR system (Applied Biosystems, Foster City, California, USA) using 384-well PCR microplates (Thermo Scientific). Reaction efficiencies for each plate were determined by generating a standard curve for each primer pair using serial dilutions of pooled first-strand cDNA templates from all ovary samples, as described by Rojo-Bartolomé et al. (2017) [5]. Gene transcription levels were standardized based on the exact cDNA quantity loaded for each sample in the RT qPCR utilizing a modified ΔCT formula according to Rojo-Bartolomé et al. (2020) [3] and Valencia et al. (2020) [34]. To enhance graphical visualization, RQ values were log (10) transformed prior to analysis.

### 2.8 Statistical analysis

All the Statistical analyses were conducted using SPSS software (Version 28, SPSS Inc., Microsoft Co., Redmond, USA). The Shapiro-Wilk test (n < 30) was employed to assess the normal distribution of the dataset and homogeneity of variances was tested using Levene’s test. Differences in tRNA/5.8S rRNA and 5S/5.8S rRNA indices, across histologically defined groups, were evaluated using the non-parametric Kruskal-Wallis test with pairwise comparison followed by Dunn’s test. Transcript levels of all target genes were also analysed using the non-parametric Kruskal-Wallis test followed by Dunn’s post hoc pairwise comparisons, as the assumptions of normality and homogeneity of variances were not satisfied. Statistical significance was set at (*p* < 0.05) for all analyses.

## 3. Results

### 3.1 tRNA and rRNAs in mullet gonad through the oogenesis

**Fig 1:**
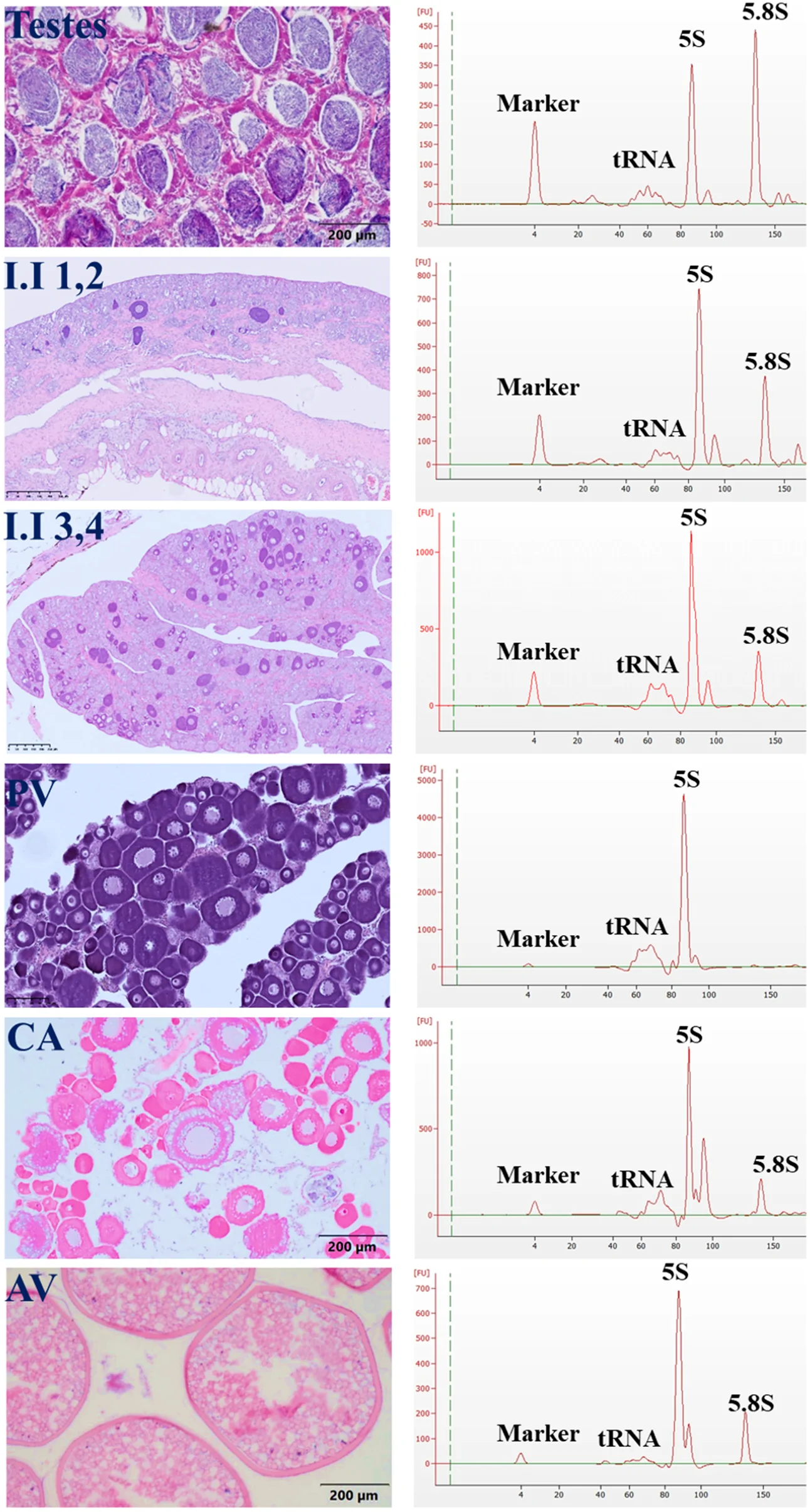
Representative small RNA electropherograms showing the relative amount of tRNAs, 5S rRNA and 5.8S rRNA in thicklip grey mullet testes, intersex testes and in ovaries at different stages of oogenesis. Micrographs alongside each electropherogram illustrate the maturity stage in each case (I.I. 1,2 = low intersex severity, I.I. 3,4 = high intersex severity, PV: previtellogenic ovaries, CA: ovaries at cortical alveoli stage, AV: advanced vitellogenic ovaries). Scale bar: Testes, I.I. 1.2, I.I. 3,4 and PV = 100 µm; CA and AV = 200 µm.

The small RNA electropherograms revealed significant differences in the RNA profiles between testes and ovaries with high levels of 5S rRNA and tRNAs in ovaries and high levels of 5.8S rRNA in testes (Fig 1). During the early stages of oogenesis (previtellogenic oocytes, PV), both tRNA and 5S rRNA levels were notably higher than those of 5.8S rRNA. However, as oogenesis progressed from cortical alveoli stage (CA) to advanced vitellogenesis (AV), the relative levels of tRNA and 5S rRNA decreased, while the proportion of 5.8S rRNA increased (Fig 1).

### 3.2 tRNA/5.8S rRNA and 5S/5.8S rRNA indices: Identification of oocyte maturation stages

**Fig 2:**
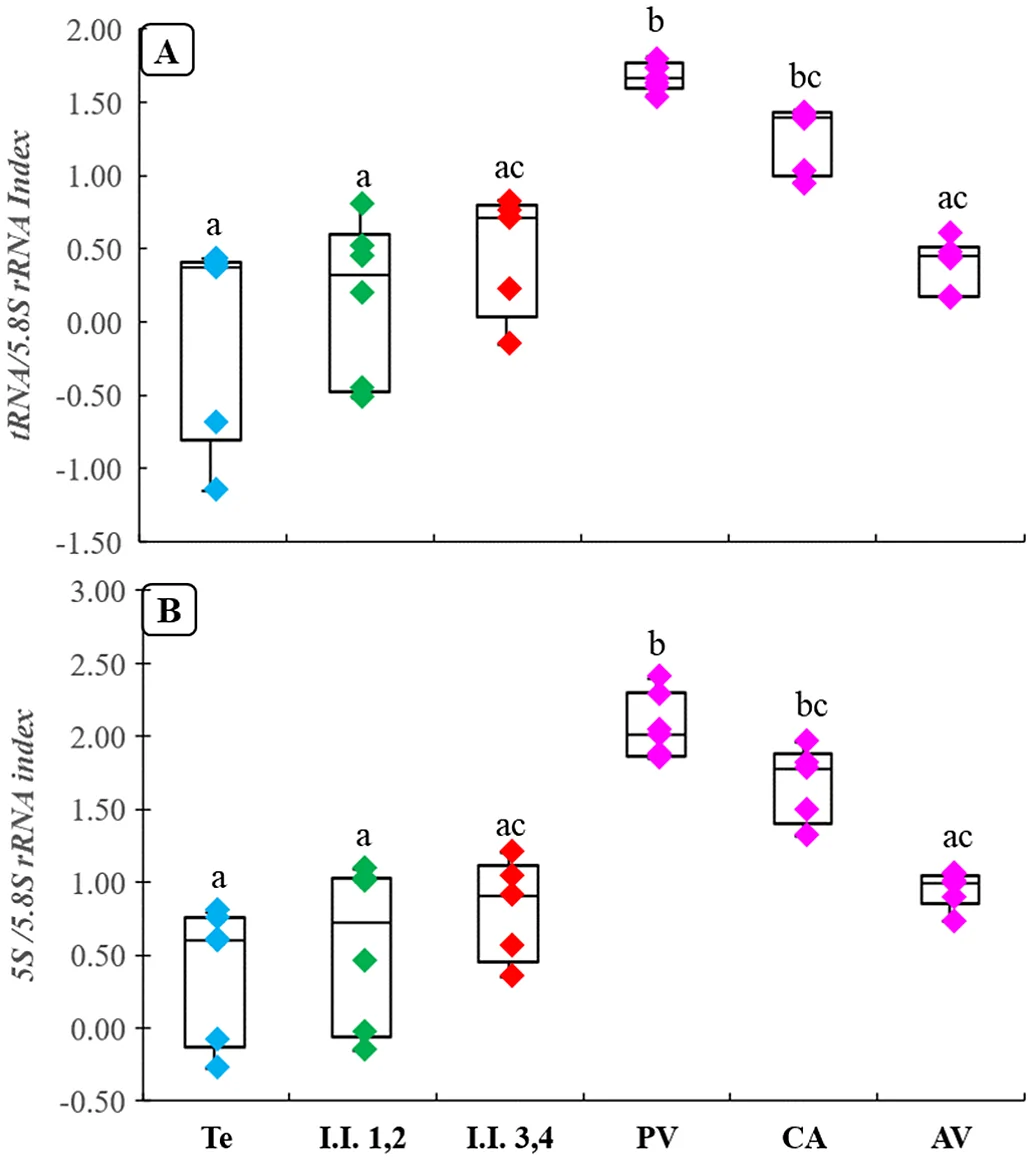
tRNA/5.8S rRNA and 5S/5.8S rRNA indices values in mullet testes, intersex testes (severity indexes I.I. 1,2 and I.I. 3,4) and ovaries at different oogenic stages (PV = previtellogenesis; CA = cortical alveoli; AV = advanced vitellogenesis). Each point on the box plots represents one individual. Boxes represent the data within the 25th and 75th percentiles; the line the median value, and top and bottom whiskers the minimum and maximum values. Different letters indicate significant differences among pairs of means (Kruskal-Wallis test followed by Dunn’s post hoc test, *p* < 0.05).

Both the tRNA/5.8S rRNA and the 5S/5.8S rRNA indices showed higher values in the ovaries than in testes (Fig 2). Index values changed across oogenesis with highest values in the ovaries at PV stage, progressively decreasing during secondary growth at the CA stage, and further during AV (Fig 2). Irrespective of their severity index no significant differences were observed between intersex individuals and testes, although some of the intersex testes displayed index values above any testis and more similar to some AV ovaries (Fig 2). Both indices provided very similar results.

### 3.3 Transcription levels in gonads of *C. labrosus*

#### 3.3.1 Transcription of sex marker genes and TFIIIA coding genes.

**Fig 3:**
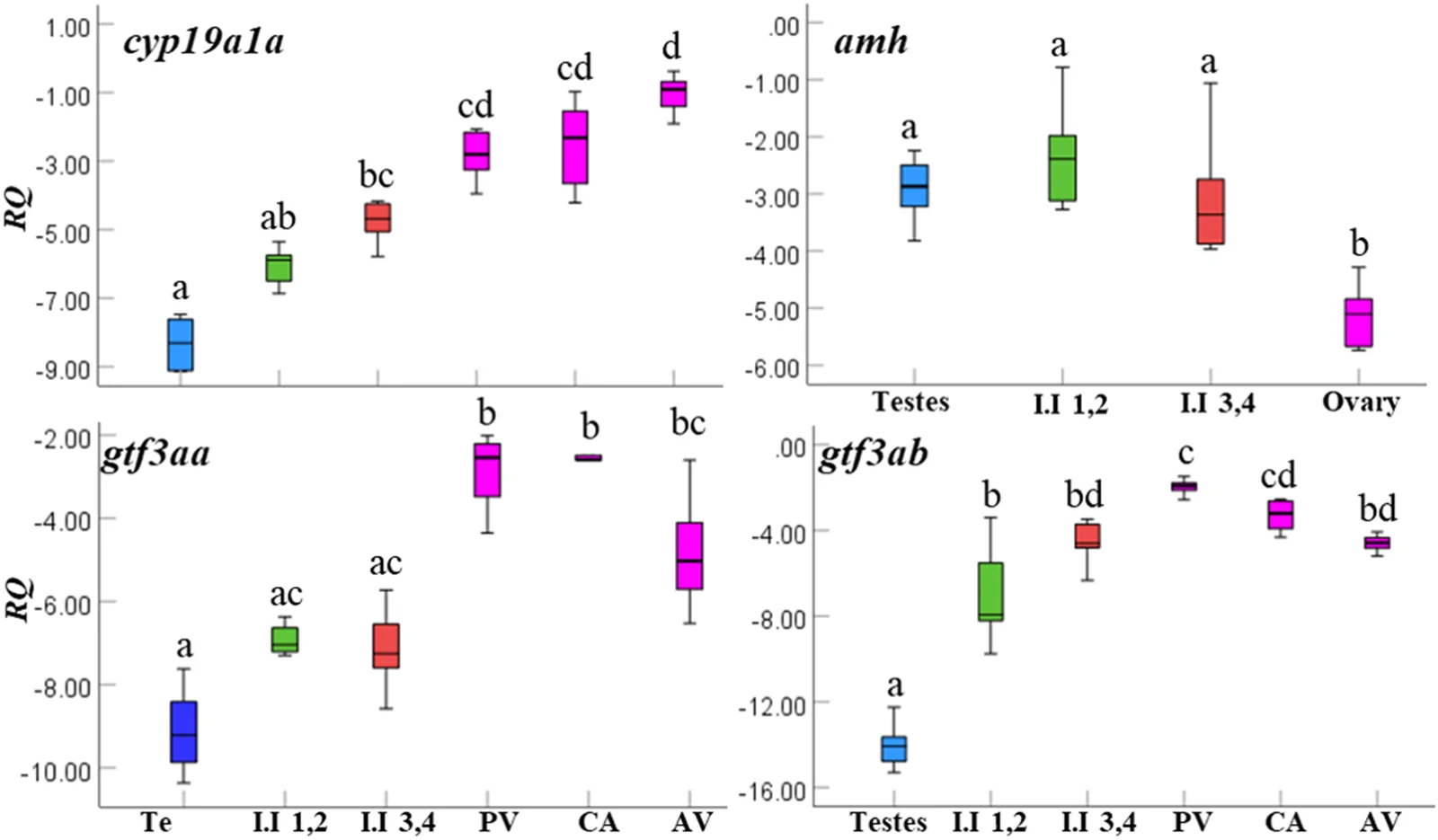
Transcription levels of sex marker and TFIIIA paralog genes in the testes (Te), intersex testes (I.I. 1,2 and I.I. 3,4) and ovaries through oogenesis from previtellogenesis (PV) to advanced vitellogenesis (AV) in thicklip grey mullets. Box plots display the 25th to 75th percentile range, with the median marked by a line, and whiskers representing the minimum and maximum values. Different letters denote significant differences among means (Kruskal-Wallis test followed by Dunn’s post hoc test, *p* < 0.05).

The sex marker genes (*cyp19a1a* and *amh)* along with TFIIIA coding mullet paralogs (*gtf3aa* and *gtf3ab*), showed distinct transcription patterns tied to gonadal development and sex. The female marker gene *cyp19a1a* is very highly transcribed in ovaries in comparison to testes, peaking in AV ovaries although without significant differences across oogenesis (Fig 3). Severe intersex individuals showed significantly higher transcription levels than normal testes. Transcription levels were statistically different in comparison to ovaries at PV and CA stages (Fig 3). The male marker gene *amh* showed significantly higher transcription levels in testes than in ovaries, no significant downregulation being observed in intersex testes (Fig 3). Meanwhile, the *gtf3aa* and *gtf3ab* genes showed significantly higher expression in ovaries than in testes where levels of *gtf3ab* transcription were close to none existing. Intersex testes showed *gtf3ab* transcription levels in between ovaries and testes (Fig 3). During oogenesis *gtf3ab* was strongly transcribed at PV stage, with a significant reduction in transcription levels towards AV stage (Fig 3).

#### 3.3.2 Transcriptional profiling of *maf1*, *dap1b* and TFIIIB related genes.

**Fig 4:**
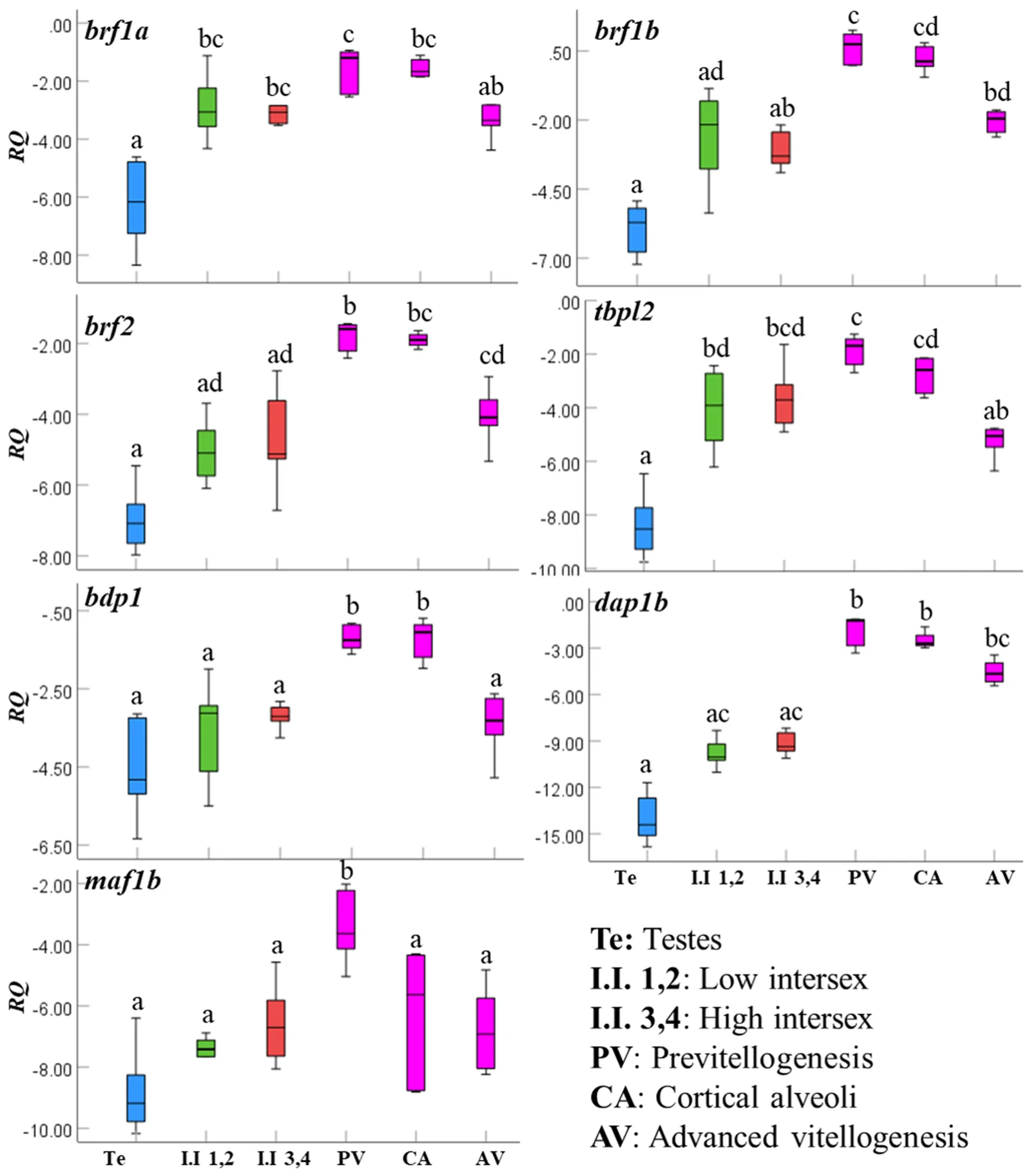
Transcription levels of TFIIIB complex coding genes and *maf1b* and *dap1b* in gonads of thicklip grey mullets. Box plots show the 25th to 75th percentile range, with the median indicated by a line, and whiskers representing the minimum and maximum values. Different letters indicate significant differences between means (Kruskal-Wallis test followed by Dunn’s post hoc test, *p* < 0.05).

TFIIIB complex coding genes (*brf1a, brf1b*, *brf2*, *bdp1* and *tbpl2*), the RNAP-III repressor *maf1b*, and the gene associated with ribosome dormancy *dap1b*, exhibit significantly higher transcription levels in ovaries than in testes, with the intersex individuals, irrespective of their severity index, showing intermediate transcription levels (Fig 4). These genes were strongly transcribed at PV stage, with progressive downregulation towards AV stage. This trend was less pronounced, although also evident, for *dap1b* (Fig 4). *maf1b* only showed significant differences in the case of PV ovaries with the highest transcription levels among all the experimental groups. Statistical differences between intersex testes and normal testes were only identified for *brf1a* and *tbpl2* (with nearly unidentifiable transcription levels in normal testes).

## 4. Discussion

In the present study, we describe the transcription pattern of genes involved in the control of ribogenesis throughout the reproductive cycle of thicklip grey mullets in relation to the pattern of accumulation of RNAP-III products, 5S rRNA and tRNAs. The tRNA/5.8S rRNA and 5S/5.8S rRNA indices molecularly differentiated ovaries along oogenesis in thicklip grey mullets in correlation to the results observed with the transcription levels of TFIIIB polypeptides coding genes *brf1a, brf1b, brf2, bdp1* and *tbpl2* and also of TFIIIA ovarian specific gene *gtf3ab*. In all cases, index and transcription levels were at their highest in previtellogenic ovaries decreasing towards advanced vitellogenesis. Always transcript levels were higher in ovaries than in testes, with intermediate levels in intersex testes, following the pattern marked by ovarian aromatase gene transcription and opposite to *amh*. The ribosomal dormancy gene *dap1b* [20] showed the same pattern of gonad transcription as the studied ribogenesis marker genes.

We have previously identified a strong expression of tRNAs and 5S rRNAs in teleost ovaries in comparison to testes and somatic tissues [1,2,4]. Our present total RNA capillary electrophoresis studies and the indices calculated thereof have revealed high levels of tRNAs in previtelleogenic ovaries that are reduced along development into the cortical alveoli stage and vitellogenesis. This reveals that the analysis of small non-coding RNAs can be easily applied to the staging of the maturation state of ovaries of synchronic developing mullets, as it was already proved with the 5S/18S rRNA index [4,35]. The indices reveal that in mullets RNAP-III products (in this case tRNAs and 5S rRNA) significantly exceed RNAP-I products (in this case 5.8S rRNA) in PV ovaries as described by Rojo-Bartolomé et al. (2016) [4] through the 5S/18S rRNA index.

In the *C. labrosus* genome (Genome assembly fCheLab1.1 in GenBank), more than 1,573 copies of the 5S rRNA gene exist, and this high copy number would suggest an evolutionary adaptation to enhance 5S rRNA production during oogenesis. In contrast, there is only one functional copy of the 45S rRNA gene in the *C. labrosus* genome [36]. This difference in gene copy number results in lower levels of 5.8S, 18S, and 28S rRNAs in the ovaries [18,37]. For zebrafish, 2330 active 5S rRNA genes tandemly repeated in chromosome 4 exist with 12 additional copies present in chromosome 18 [38,39]. This arrangement together with the transcriptional regulation by different RNAPs and specific compartmentalization of transcription, nucleoplasm for 5S rRNA vs nucleolus for 45S rRNA, would explain the differential transcription levels of both rDNA genes required for ribosome assembly. This would also modulate the dynamics of transcription along oogenesis, while the structure of chromatin and nucleoli vary as meiosis progresses [6]. In this sense, the region in which the 45S rRNA genes are present in zebrafish, also in chromosome 4, undergoes massive extrachromosomal proliferation during meiosis [39].

tRNA genes in teleost genomes have also undergone a remarkable multiplication process through which multiple redundant tRNA genes (isodecoders) exist allowing all possible codon-anticodon interactions, 61 in total [36,40]. This is not the case in mammal genomes with only 47 and 48 tRNA anticodons among the tRNA genes present in the mouse and human genomes [40–42]. In the *C. labrosus* genome (fCheLab1.1) 1,540 tRNA genes can be found, with for instance 143 different isodecoders specific to serine and 72 to alanine. Similarly, the zebrafish genome exhibits an extraordinary proliferation of tRNA genes, with approximately 15,204 copies, (i.g 573 alanine-specific isodecoders). Most of these genes are placed in the same chromosome 4 where most of the 5S rRNA and the maternal 45S rRNA genes are placed [43]. This amplified collection of tRNA genes highlights the evolutionary significance and functional complexity of protein synthesis regulation in fish genomes [37,43].

The huge accumulation of 5S rRNA in teleost ovaries has been observed to be associated to strong upregulation of *gtf3ab* [2,3]. *gtf3ab* has already been established as a marker of intersex condition in fish exposed to xenoestrogens and as a marker of presence of oocytes in general [2,4,5,25]. The present RT qPCR analysis revealed distinct transcription patterns of TFIIIB polypeptide coding genes and *maf1* through oogenesis in thicklip grey mullets with profiles mimicking those of *gtf3ab* and ovarian aromatase transcription and of RNA indices. TFIIIA is necessary exclusively for transcription of 5S rRNA genes by RNAP-III, and teleost genomes present two paralog genes, *gtf3aa* and *gtf3ab* [3]. *gtf3ab* was demonstrated to be a gene nearly exclusively transcribed in teleost ovaries [3] and as it functions also as a 5S rRNA binding protein for its storage in the cytosol in the form of 7S ribonucleoprotein [12], it is very strongly transcribed in previtellogenic ovaries and in intersex testes. As oocyte marker presently showed the same pattern of expression of the ovarian aromatase and opposite to that of the testis-specific *amh* [44,45]. A single cell transcriptomics atlas constructed for Chinese tongue sole ovaries revealed that the transition from mitotic oogonia to oocytes is marked by the upregulation of 62 genes, two of them being transcription factors, one precisely *gtf3ab* [46]. Authors reported in their enrichment analyses that the transition early oogonia to mitotic oogonia was marked by downregulation of RNA and rRNA processing among other functions. Then, the transition to primary previtellogenic was marked by the upregulation among others of ribosome biogenesis [46].

The rest of RNAP-III coding genes, those producing tRNAs, U6 snoRNA, RnaseP, 7SK RNAs and some miRNAs require other transcription factors, TFIIIB and TFIIIC and we wanted to analyse whether they behaved transcriptionally as *gtf3ab*, tRNA and 5S rRNA do. Indeed, the genes coding all TFIIIB forming peptides (*brf1a*, *brf1b, brf2, bdp1 and tbpl2*), and *maf1b* coding the main RNAP-III inhibitor protein, exhibited significantly higher transcription levels in ovaries and intersex testes than in testes, with transcript levels peaking in PV ovaries gradually decreasing towards advanced vitellogenesis. In the genome of the *C. labrosus*, as in the rest of teleosts, two *brf1* paralogs (*brf1a* and *brf1b*) exist apparently arisen after the teleost specific genome duplication event. Both paralogs displayed similar transcription patterns, with higher transcription levels in ovaries than in other organs [11]. In fish, functional information on *brf1* is limited; however, zebrafish double knockouts of *brf1a* and *brf1b* exhibit neurodevelopmental defects associated with impaired RNAP-III mediated transcription [47]. The elevated ovarian expression, specially at previtellognesis, detected here likely reflects their involvement in RNAP-III activation during teleost oogenesis together with the need to produce high levels of tRNAs. In mammals and amphibians, RNAP-III transcribed genes with type-III promoters interchange Brf1 for Brf2. Teleost genomes contain a single *brf2* ortholog, whose transcription levels across tissues resemble those of *brf1a* and *brf1b* [11]. The same was observed here across oogenesis, with strongest transcription at previtellogenesis. In zebrafish, knocking-down *brf2* also results in neurodevelopmental impairments as for *brf1* paralogs, though its gonadal role remains uncharacterized [48].

Bdp1 is a protein within TFIIIB that facilitates TFIIIC binding to the RNAP-III initiation complex, playing a critical role in the assembly and activation of RNAP-III–dependent transcription [49,50] and it presents a single paralog in thicklip grey mullets [11]. Hereby, its pattern of transcription was showing similar profiles to those of the other studied genes, but without significant differences between intersex testes and testes. However, it should be reminded that plenty of teleost species, although not in the case of euteleosts, present two *bdp1* paralog genes [11], and in such species the transcriptional activity of each paralog could be different to the one identified here.

All vertebrates possess three TATA-binding protein coding genes, with the vertebrate-specific paralog *tbpl2* showing predominantly gonadal expression, also in teleosts were transcription has been identified mainly in ovaries [11,51,52]. *tbpl2* seems therefore a potential marker of oocyte differentiation under both physiological and exposure to xenoestrogens conditions leading to intersex phenotypes as observed presently. We did not analyse the pattern of transcription of the other two mullet TATA-binding protein coding genes.

Maf1, is a conserved transcriptional repressor of RNAP-III first identified in *S. cerevisiae*, and conserved across eukaryotes [7,13,16] (*C. labrosus*, as most euteleosts has lost the second *maf1* paralog gene present before the appearance of teleosts in chondrichthyans and spotted gar, and keeps only *maf1b*. [11]. The pattern of transcription observed was similar to that *bdp1* but, as for this gene, it must be remembered that other non-euteleost fish (such as *Danio rerio*) do conserve the second paralog *maf1a*, that could behave differentially.

This general pattern of gene transcription and non-coding RNA expression points to a strong production of ribosomes in thicklip grey mullet ovaries, specially during previtellogenesis. This is also the case of intersex testes, which only exceptionally present oocytes in secondary growth [25]. If intersex severity index is high this is still more evident as more oocytes are present in testis. This ribosomes would in principle be produced maternally for utilization in the future embryo, so during oocyte development they should be maintained in a pre-assemble stage or somehow dormant. Recent research has highlighted Dap1b*,* together with other three proteins, namely elF5A, Habp4 and eEF2, as a crucial modulator of ribosomal dormancy by binding with the maternal ribosomes in the zebrafish ooplasm [19,20]. Dap1b would inhibit translation capacity of the ribosomes by stably inserting into the polypeptide exit tunnel of the ribosome [20]. Dap1b would thus be fundamental for regulating and safeguarding the precise timing of protein synthesis during the critical oocyte-to-embryo transition. Here we only analyzed *dap1b* gene transcription levels and observed a strong transcription in ovaries resembling the transcription pattern identified for the previously mentioned genes across oogenesis. However, *dap1b* transcript levels were maintained quite constant along oogenesis without a significant downregulation during advanced vitellogenesis. A trend towards upregulation was also observed in intersex testes in comparison to testes. These transcriptional dynamics highlight the interplay of sex-specific and stage-specific gene regulation during fish gametogenesis. The evaluated molecular markers are easy and fast to quantify can be employed to classify ovaries according to their maturation stage.

Rapid cleavage divisions and the transition from maternal to zygotic control of gene expression mark early embryonic development in most species [53]. Early development in fish is characterized by rapid cell division with nearly non-existing interphases. Maternal mRNAs, proteins, RNAs, but also ribosomes loaded maternally along oogenesis are essential to drive these rapid early divisions. The maternal to zygotic transition in zebrafish occurs around the 10th cell division, around 3 hours post fertilization and it is at this moment that the embryo shifts from relying on maternally supplied macromolecules to the activation of its own genes [54] and production of its own ribosomes.

## 5. Conclusions

Overall, the results presented here confirm that RNAP-III transcripts, tRNAs, as it was already proved for 5S rRNA, together with the indices that can be calculated from their capillary electrophoretic analyses are valuable molecular markers to identify the oogenic stage of synchronously developing thicklip grey mullet ovaries. RNAP-III activity is regulated in eukaryotic cells by general transcription factors whose coding genes *gtf3ab* in the case of 5S rRNA genes and *brf1a*, *brf1b*, *brf2*, *bdp1* and *tbpl2* show a transcription profile that correlated with the pattern displayed along oogenesis by the tRNA/5.8S rRNA and the 5S/5.8S rRNA index. All these results point to the importance of regulating ribogenesis and protein synthesis capacity at the beginning of oogenesis, in deep contrast with spermatogenesis. This would prepare the oocyte for fertilisation and for the early protein production needs during initial embryo development. In this sense, *dap1b* that according to Leesch and colleagues [20] codes a protein that can bind ribosomes to keep them in hibernation showed very strong transcription levels in ovaries in comparison to testes. Efforts are currently underway to develop a fish sexing and fish oogenesis staging molecular kit based on total RNA capillary electrophoresis analysis and RT qPCR assessment of transcript levels, which could have significant potential for technological transfer in fisheries stock management and environmental biomonitoring of xenoestrogenicity. Further studies across different fish species and environmental conditions are needed to understand the mechanisms of ribogenesis during oogenesis in teleosts, as metazoan models to analyse the regulation of protein synthesis under physiological and stress conditions.
